# Recent Advances in Preservation Techniques for Edible and Medicinal Mushrooms

**DOI:** 10.3390/foods15132328

**Published:** 2026-07-01

**Authors:** Sunčana Včelik, Anita Pichler, Nela Nedić Tiban, Drago Šubarić, Tihomir Kovač

**Affiliations:** Faculty of Food Technology Osijek, Josip Juraj Strossmayer University of Osijek, Franje Kuhača 18, 31000 Osijek, Croatia; svcelik@ptfos.hr (S.V.); apichler@ptfos.hr (A.P.); nela.nedic@ptfos.hr (N.N.T.); dsubaric@ptfos.hr (D.Š.)

**Keywords:** bioactive compounds, edible and medicinal mushrooms, innovative preservation technologies, postharvest preservation, shelf-life extension

## Abstract

Edible and medicinal mushrooms, including cultivated and wild species, are increasingly recognized as valuable functional foods and nutraceutical resources due to their high nutritional value, abundance of bioactive compounds, and documented health-promoting properties. However, their high perishability results in substantial postharvest quality losses and limits commercial shelf life. This review provides a comprehensive overview of recent advances in mushroom preservation technologies, with particular emphasis on emerging non-thermal approaches such as cold plasma treatment, active packaging systems, and electrostatic field technologies. Conventional and advanced drying methods, edible coatings, biopreservation, fermentation and irradiation are also critically evaluated. Cold plasma treatment effectively reduces microbial contamination and enzymatic browning while maintaining firmness and nutritional quality, whereas active packaging systems based on chitosan films, nanocomposites, and modified atmospheres help reduce moisture loss, delay senescence, and preserve physicochemical properties during storage. Electrostatic field treatment combined with modified atmosphere packaging has shown additional potential for extending refrigerated shelf life. Among drying technologies, freeze-drying generally provides the highest retention of colour, texture and bioactive compounds, although its industrial application remains constrained by high energy consumption and operational costs. Overall, current evidence suggests that integrated preservation approaches offer the greatest potential for improving shelf-life extension and quality retention. Nevertheless, further research is required to address challenges related to industrial scalability, process standardization, economic feasibility and long-term quality assessment.

## 1. Introduction

**Figure 1:**
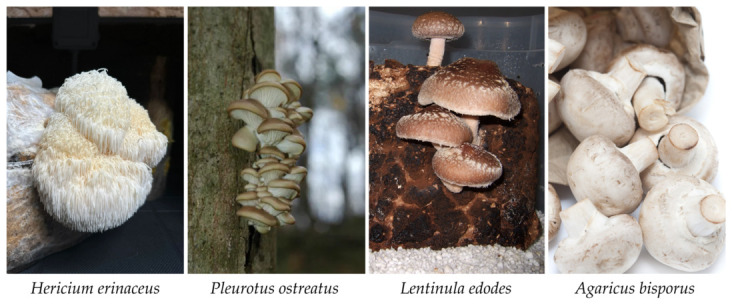
Overview of selected edible and/or medicinal mushrooms.

Edible and medicinal mushrooms are increasingly recognized as functional foods, owing to their high nutritional value, abundance of bioactive compounds and wide range of therapeutic effects [1]. These fungi are nutrient-dense foods that exhibit antimicrobial, antioxidant and anticancer activities [2,3,4]. Major commercially cultivated edible mushrooms discussed in the reviewed literature include *Agaricus bisporus*, *Lentinula edodes* and *Pleurotus* spp., while the broader literature on edible and medicinal mushrooms also encompasses wild species [5,6,7,8]. An overview of the representative edible and/or medicinal mushroom species discussed in this review is presented in Figure 1. Despite their nutritional and economic importance, fresh mushrooms are among the most perishable agricultural commodities, resulting in rapid quality deterioration and substantial postharvest losses [1,4,9]. Quality deterioration manifests as weight loss, texture softening, surface browning, loss of whiteness and degradation of bioactive compounds [10,11]. The global edible mushroom industry has expanded considerably over recent decades. Global edible fungi production reached approximately 50 million tonnes in 2023, while the global market was valued at USD 33.16 billion in 2024. Despite this growth, mushrooms remain highly perishable because of their high moisture content and continued postharvest respiration. Approximately 10% of edible fungi in the Chinese market are discarded due to quality losses, highlighting the need for effective preservation strategies [12,13]. Recent advances in preservation technologies have focused on non-thermal and minimally invasive approaches that maintain nutritional quality while extending shelf life, including cold plasma treatment and advanced packaging systems [4,9]. This comprehensive review synthesizes current knowledge on modern preservation techniques for edible and medicinal mushrooms, with particular emphasis on cold plasma treatment, active packaging, antimicrobial photodynamic therapy and electrostatic processing, while also examining advanced drying technologies, edible coatings, biopreservation strategies and irradiation methods. Recent literature has predominantly addressed individual storage strategies or selected preservation approaches for mushrooms. Consequently, the present review integrates both conventional and emerging preservation technologies, with particular attention to cold plasma, active packaging systems, antimicrobial photodynamic treatment, electrostatic processing, drying methods, edible coatings, biopreservation and irradiation. The review further examines their effectiveness in extending shelf life, maintaining product quality, practical implementation and future research opportunities.

## 2. Background and Theoretical Foundations

### 2.1. Mushroom Perishability and Quality Deterioration Mechanisms

**Figure 2:**
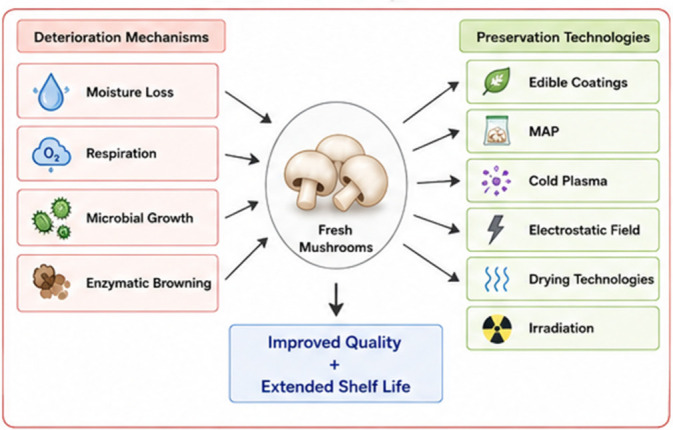
Deterioration Mechanisms and Preservation Technologies.

Fresh mushrooms are among the most perishable agricultural commodities, with shelf life typically limited to 1–3 days at room temperature and 8–10 days under refrigerated conditions [1]. Their high moisture content (85–95%), lack of a protective cuticle, continuous postharvest metabolic activity, and susceptibility to enzymatic browning and microbial contamination contribute to rapid quality deterioration during storage [4,9]. The extreme perishability of fresh mushrooms results from a complex interaction of physiological, biochemical and microbiological factors [1,4,14]. The major deterioration mechanisms together with the principal preservation technologies discussed in this review are summarized in Figure 2. Unlike most fruits and vegetables, mushrooms lack a protective cuticle layer, making them highly susceptible to moisture loss through transpiration. Their thin and porous epidermal structure facilitates rapid water migration, resulting in significant postharvest weight loss and quality deterioration [1,11]. Enzymatic browning represents a major quality deterioration mechanism in mushrooms, mediated primarily by polyphenol oxidase (PPO), which catalyzes the oxidation of phenolic compounds to quinones that subsequently form brown pigments [11,15]. This browning reaction is accelerated by mechanical damage, exposure to oxygen and elevated temperatures. A recent study investigating postharvest preservation of *Agaricus bisporus* has shown that plasma-activated water produced using dielectric barrier discharge (DBD) cold plasma can effectively suppress polyphenol oxidase activity during refrigerated storage. Under treatment conditions of 50 kV for 20 min using air as the carrier gas, plasma-activated water (PAW)-treated mushrooms exhibited markedly lower PPO activity compared with untreated controls. PPO values declined from 1788 U/min in untreated samples to 228 U/min after 5 days of storage at 4 °C, indicating a strong inhibitory effect of the treatment on enzymatic browning processes [15]. Respiratory metabolism continues post-harvest, consuming stored carbohydrates and generating heat, carbon dioxide and water vapour [3,9]. High respiration rates accelerate senescence and quality loss. Modified atmosphere packaging (MAP) and electrostatic field treatments have shown promise in suppressing respiratory intensity and extending shelf life [10,16]. Microbial contamination by bacteria, yeasts and fungi contributes to quality deterioration and food safety concerns [1,11,17]. The high moisture content and lack of a protective cuticle in mushroom tissue provide favourable conditions for microbial growth and rapid spoilage. Cold plasma treatment has emerged as an effective non-thermal preservation technology, reducing total viable counts by approximately 16.5% while maintaining important quality attributes such as colour, firmness, vitamin C and protein content in mushroom tissue [11].

### 2.2. Nutritional and Medicinal Significance

Edible and medicinal mushrooms are recognized as functional foods with exceptional nutritional and therapeutic properties [1,14,18]. They are rich sources of high-quality protein (20–40% dry matter), dietary fibre, vitamins (particularly B-complex vitamins), minerals (selenium, potassium, phosphorus) and bioactive compounds including polysaccharides, phenolic compounds, terpenoids and ergosterol [1,19,20]. The medicinal significance of mushrooms is primarily attributed to β-glucans and other polysaccharides that exhibit immunomodulatory, antitumor, antioxidant and antimicrobial activities [18,21]. *Hericium erinaceus* demonstrates neuroprotective properties, while *Lentinus edodes* contains lentinan, a β-1,3-glucan with documented anticancer effects [18]. Wild edible species such as *Boletus edulis* and *Cantharellus cibarius* are particularly rich in phenolic compounds and exhibit strong antioxidant activities [21]. Preservation methods must balance shelf-life extension with retention of nutritional and bioactive compounds. Freeze-drying is considered one of the most effective techniques for preserving heat-sensitive compounds and maintaining mushroom quality, particularly regarding texture, microstructure, vitamins, phenolic compounds and antioxidant activity, although low-temperature hot-air drying may achieve comparable results in some cases [19,20,22]. However, the high energy consumption and operational costs associated with freeze-drying have prompted the exploration of alternative preservation strategies capable of achieving comparable quality retention with improved energy efficiency and lower processing costs [3,23].

## 3. Modern Preservation Methods and Technologies

### 3.1. Cold Plasma Treatment

**Figure 3:**
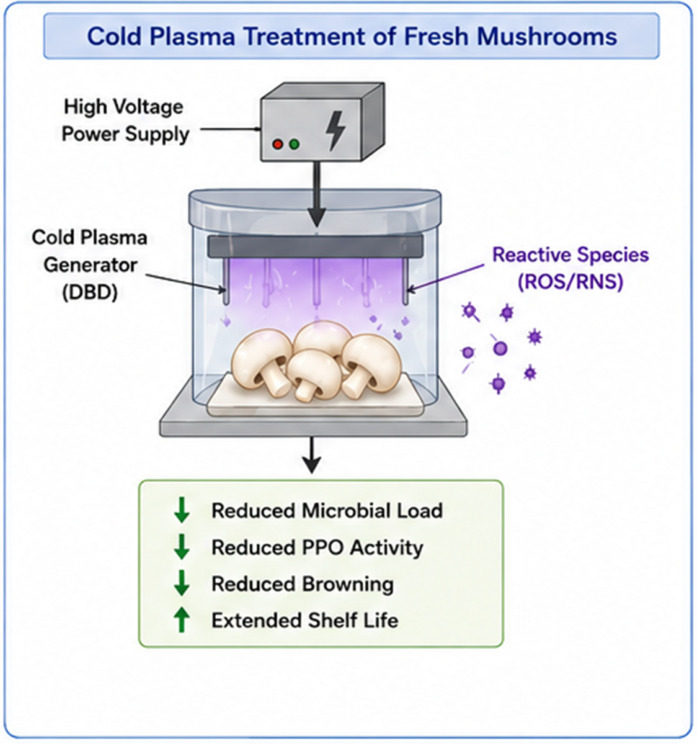
Cold Plasma Treatment System.

Cold plasma treatment has emerged as a promising non-thermal preservation technology for fresh mushrooms, offering effective microbial decontamination while maintaining quality attributes [15,24]. Cold plasma is a partially ionized gas rich in reactive oxygen and nitrogen species (ROS/RNS), ions, electrons and free radicals that exhibit antimicrobial activity by inducing membrane damage, lipid peroxidation, enzyme inactivation and DNA degradation [11,15]. Cold plasma can be applied through two primary approaches: direct plasma gas treatment and PAW immersion. Direct plasma treatment involves exposing mushroom surfaces to plasma-generating equipment such as DBD systems, whereas PAW treatment involves immersing mushrooms in water previously activated by plasma exposure, allowing reactive species to be transferred into the aqueous medium [15]. A schematic overview of the cold plasma treatment system and its principal application modes is presented in Figure 3. Reported effects of cold plasma may vary depending on the treatment medium and the reactive species generated. Recent studies comparing cold argon plasma, plasma-activated air and plasma-activated water demonstrated differences in microbial inactivation and quality preservation, with plasma-activated water generally showing the highest preservation efficacy. Although inhibition of polyphenol oxidase (PPO) activity has been associated with plasma-generated reactive oxygen and nitrogen species, the precise molecular mechanisms responsible for PPO suppression remain insufficiently understood and require further investigation [25]. Recent research on *Agaricus bisporus* demonstrated that cold plasma treatment under optimal conditions (95 kV, 130 Hz, 10 min) reduced total viable counts by approximately 16.5% while preserving superior quality attributes, including lower browning and higher firmness during storage [11]. The treatment effectively inhibited enzymatic browning, with PAW showing particularly strong efficacy in reducing the browning index (BI) and ΔE colour values during storage. PAW treatment inhibited PPO activity, decreasing it from 1788 U/min in control samples to 228 U/min after 5 days of storage, corresponding to an approximately 87% reduction in PPO activity [15]. Cold plasma treatment improved the postharvest quality of *Agaricus bisporus*, with treated mushrooms showing 26.9% lower browning levels and 25.6% greater hardness than untreated controls. The treatment also preserved vitamin C and total protein content more effectively than conventional storage methods [11]. For *Flammulina velutipes* (enoki mushrooms), cold plasma treatment improved postharvest storage quality by reducing microbial growth, delaying vitamin C loss and browning, maintaining cell membrane integrity and enhancing antioxidant enzyme activities during refrigerated storage [26]. Cold plasma technology, particularly in the form of plasma-activated water PAW, demonstrated strong potential for preserving mushroom texture and minimizing postharvest dehydration. PAW-treated *Agaricus bisporus* maintained superior firmness (19.69 N after 5 days of storage) and lower weight loss (0.15% after 3 days) compared with untreated controls, indicating delayed senescence and improved storage stability [15]. Cold plasma pretreatment has shown considerable potential for improving subsequent hot-air drying processes. When applied prior to hot-air drying of shiitake mushrooms, cold plasma induced microstructural surface modifications, including increased intracellular spaces and cell wall disruption, which facilitated faster and more efficient drying compared with untreated controls [24,27]. Cold plasma pretreatment enhanced the physicochemical properties of mushroom powder, including bulk density, water retention capacity and swelling index. Moreover, it simultaneously improved the retention of nutritional compounds during hot-air drying, such as sugars, vitamins, or phenolic acids, and preserved higher antioxidant activity compared with untreated hot-air-dried samples [24]. This synergistic approach demonstrates the potential for integrating cold plasma technology with traditional preservation methods to achieve enhanced quality outcomes.

### 3.2. Active Packaging Systems

Active packaging represents an advanced preservation strategy that extends beyond the passive barrier role of conventional packaging by actively interacting with the packaged food and/or the headspace atmosphere to maintain quality, improve safety and prolong shelf life [9,28,29]. For mushrooms, active packaging systems have evolved in recent years, incorporating antimicrobial agents, oxygen scavengers, moisture regulators and gas-permselective materials to extend shelf life as well as maintain postharvest quality [9,28]. MAP remains a cornerstone technology for fresh mushroom preservation, involving the modification of the gaseous environment surrounding the product to slow respiration, inhibit microbial growth and delay senescence [10,16,30]. The optimal gas composition varies by species, although MAP systems commonly use elevated CO_2_ concentrations (approximately 5–20%) combined with reduced O_2_ levels to slow respiration and senescence [9,16]. Research on *Pleurotus sapidus* (oyster mushroom) evaluated three active MAP treatments: high carbon dioxide packaging (HCP), low carbon dioxide packaging (LCP) and high nitrogen packaging. HCP demonstrated superior preservation performance, yielding the highest total phenolic content (3.03 mg GAE/g) and the highest lightness L* value (60.36) after ten days of storage. Sensory evaluation indicated that HCP and LCP were the most effective treatments for maintaining mushroom colour and odour quality. HCP also achieved the most favourable Total Quality Index, with values of 3.44 at day 5 and 4.74 at day 10. However, these findings are based on a single study and additional investigations are required to confirm the broader applicability of HCP for mushroom preservation [16]. For *Agaricus bisporus*, MAP enhanced shelf life by controlling weight loss, maintaining firmness and delaying cap opening and browning during refrigerated storage [30]. Modified chitosan-based films and coatings have gained considerable attention as active packaging materials due to chitosan’s inherent antimicrobial, antioxidant and film-forming properties. These biopolymer-based materials enhance mushroom preservation by slowing respiration, inhibiting microbial growth, protecting antioxidant compounds and regulating enzyme activity. The incorporation of functional additives into chitosan matrices further improves preservation efficacy. Essential oils and polyphenolic compounds enhance antioxidant and antimicrobial properties, while additional modifications improve water and gas barrier properties and reduce light transmittance [28]. Research has demonstrated that chitosan-guar gum composite edible coatings effectively preserve *Lentinus edodes* quality during postharvest storage, though specific quantitative outcomes vary with formulation and application methods [31]. For *Agaricus bisporus*, various polysaccharide-based edible coatings including polysaccharide gum, agar, sodium alginate, egg white protein and lecithin have been evaluated [32]. Sodium alginate-based edible coatings have demonstrated promising postharvest preservation effects in edible mushrooms. Furthermore, advanced packaging technologies increasingly incorporate nanomaterials and biodegradable polymers to enhance preservation efficacy while addressing environmental sustainability concerns [9,29]. Nanocomposite packaging offers improved mechanical properties, enhanced barrier characteristics, and enables the incorporation and controlled delivery of antimicrobial agents [9]. Poly(L-lactic acid)-based soft films with gas permselectivity represent an innovative approach for *Agaricus bisporus* preservation. These biodegradable films can be synthesized through one-step processes and offer tailored gas transmission properties that optimize the internal atmosphere while maintaining structural integrity [29]. The integration of active, biodegradable and nano-based materials represents a growing trend in mushroom packaging research [9]. Combined packaging systems that leverage multiple functional components show enhanced preservation efficacy compared to single-component approaches, though commercial implementation remains limited by cost and regulatory considerations [9]. Active edible coatings that incorporate antimicrobial and antioxidant compounds represent a hybrid approach combining the benefits of edible films with active preservation agents [33,34]. Research on oyster mushrooms (*Pleurotus ostreatus*) demonstrated that lipid-based edible coatings containing glycerol monostearate (GMS) combined with *Thymus vulgaris* extract (TE) at 150 mg/kg improved the preservation of postharvest quality attributes [33]. The GMS + TE150 coating resulted in high texture tightness, low weight loss and minimal changes in colour indices (a*, b*, L*). The combination showed strong antimicrobial effects and maintained mushroom brightness and appearance throughout storage. The GMS coating reduced weight loss by obstructing surface pores and limiting oxygen penetration, while thyme extract enhanced the antimicrobial and antioxidant properties due to its phenolic compounds [33]. An innovative preservation approach combining an active edible coating based on *Salvia macrosiphon* seed gum enriched with liquid smoke and Ultraviolet B (UV-B) irradiation demonstrated synergistic effects in preserving button mushrooms. This multi-hurdle strategy combines the antimicrobial and antioxidant properties of the coating components with UV-B treatment to enhance postharvest quality and extend shelf life [34].

### 3.3. Emerging Non-Thermal Technologies

#### 3.3.1. Electrostatic Field Technology

**Figure 4:**
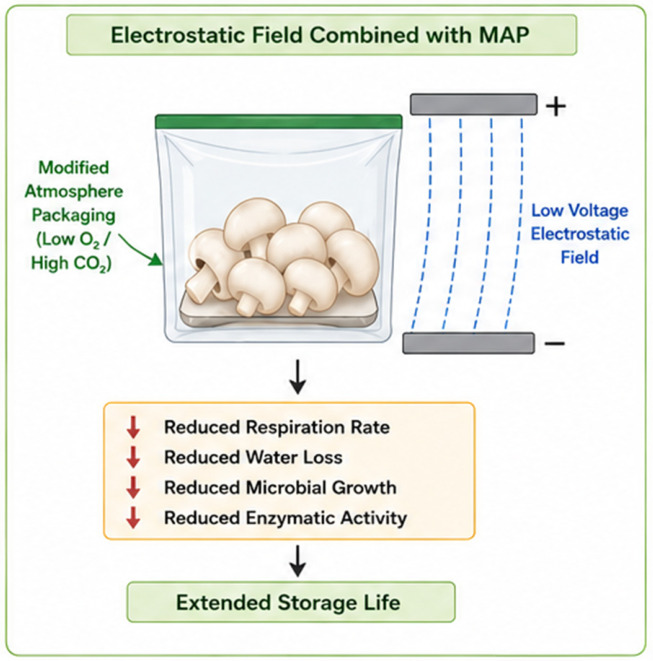
Combined electrostatic field and MAP preservation system.

Low-voltage electrostatic field (LVEF) combined with MAP has emerged as a promising non-thermal preservation strategy for fresh mushrooms. In *Agaricus bisporus*, the combined LVEF–MAP treatment suppressed respiration intensity, limited microbial proliferation, regulated enzyme activity, and helped preserve membrane stability during refrigerated storage. In addition, MAP reduced moisture loss, while LVEF contributed to lower CO_2_ levels compared with MAP alone. Owing to these synergistic effects, the storage life of button mushrooms was prolonged from approximately 6 days to more than 12 days at 4 °C, indicating considerable potential for postharvest mushroom preservation [10]. Figure 4 illustrates the combined electrostatic field and modified atmosphere packaging (MAP) preservation system used for postharvest mushroom storage. High-voltage electric field (HVEF) treatment has also been investigated as a non-thermal preservation approach for *Agaricus bisporus*. During refrigerated storage, HVEF helped maintain mushroom firmness and whiteness, reduced lipid peroxidation, preserved phenolic compounds and enhanced antioxidant enzyme activity. In addition, HVEF suppressed PPO activity and contributed to better preservation of mushroom microstructure, thereby delaying browning and senescence during storage [35].

#### 3.3.2. Antimicrobial Photodynamic Therapy

Although antimicrobial photodynamic therapy (aPDT) has emerged as a promising non-thermal microbial control technology, research specifically focused on mushroom preservation remains limited. Current studies mainly investigate UV-based preservation approaches. In *Agaricus bisporus*, UV-B irradiation combined with active edible coatings based on *Salvia macrosiphon* seed gum and liquid smoke demonstrated synergistic preservation effects by reducing microbial growth, delaying browning and softening; thus maintaining sensory quality and phenolic content during storage. UV-B treatment also increased vitamin D_2_ content, highlighting its additional functional benefits for mushroom preservation [34]. Although aPDT has shown promising antimicrobial effects in food preservation systems, studies directly evaluating its application in edible mushrooms remain limited. Therefore, evidence from UV-based treatments and related photodynamic approaches is discussed as an indirect indication of the potential applicability of aPDT in mushroom preservation.

### 3.4. Advanced Drying Technologies

Drying remains the most widely used preservation method for mushrooms, reducing water activity to levels that inhibit microbial growth and enzymatic reactions while enabling long-term storage and facilitating transportation [36,37,38]. Over the past years, significant advances in mushroom drying technologies have focused on improving energy efficiency, preserving product quality and developing hybrid drying approaches that combine multiple drying methods [3,39].

#### 3.4.1. Freeze-Drying (Lyophilization)

Freeze-drying (FD), or lyophilization, consistently demonstrates superior quality retention compared to thermal drying methods and is widely regarded as one of the most effective techniques for preserving high-value mushroom products [19,36,37]. The process involves freezing the product followed by sublimation of ice under vacuum, avoiding the liquid phase and minimizing thermal degradation of heat-sensitive compounds [19,36]. Research on *Stropharia rugosoannulata* demonstrated that optimized vacuum FD resulted in products with bright colour, minimal browning, maintained natural white colour and intact internal structure retaining natural form [36,40]. Vacuum FD enabled high retention of soluble proteins in *Stropharia rugosoannulata*, with concentrations reaching approximately 68 mg/g. The optimized drying conditions also contributed to improved preservation of cellular structure and biologically active compounds compared with conventional drying approaches [40]. In *Pleurotus eryngii*, vacuum FD resulted in the lowest shrinkage rate (30.2%), the highest rehydration ratio (3.03) and the greatest retention of polysaccharides (13%) compared with the other drying methods evaluated [23]. Comparative studies on *Boletus edulis* demonstrated that FD best preserved positive sensory attributes, whereas convective drying at 70–80 °C retained the highest concentrations of volatile compounds and maintained strong mushroom aroma characteristics [39]. For *Lyophyllum decastes*, FD achieved the best colour retention, highest rehydration capacity (5.66) and minimal tissue damage, with flavour profiles most similar to fresh samples [41]. Studies on *Oudemansiella raphanipes* showed that vacuum freeze-drying (VFD) resulted in minimal collapse, mild shrinkage, uniform porous microstructure, the highest L value (67.09) indicating superior colour retention and the highest 5′-nucleotide content (2.44 mg/g), while vacuum microwave drying (VMD) produced the highest crude protein content (26.13 mg/g) [37]. However, FD has significant limitations, including long drying times (19–22 h or longer), high energy consumption (e.g., 6.67 kWh/kg for shiitake mushrooms), low drying efficiency, and consequently high operational costs [3,39,41]. These economic and operational constraints limit the industrial application of FD mainly to high-value products and encourage the development of alternative drying technologies capable of achieving comparable product quality with lower energy consumption and processing costs [2,3].

#### 3.4.2. Hot Air Drying and Thermal Methods

Hot air drying (HAD) is widely used in commercial mushroom processing because it combines relatively low processing costs with satisfactory preservation of product quality compared with more advanced drying technologies such as FD [39,42,43]. However, thermal drying methods inevitably cause some quality degradation due to heat exposure, including colour changes, texture alterations, volatile compound losses and degradation of heat-sensitive nutrients [3,39]. Optimizing drying temperature is essential to maintain product quality while ensuring efficient processing. In *Pleurotus sajor-caju*, hot-air drying at 40 °C resulted in the highest protein retention (30.81%), while biochemical parameters such as ascorbic acid, antioxidant activity and total phenolic content remained comparable to freeze-dried samples. Considering both product quality and processing costs, this drying condition was identified as the most suitable option [20]. In *Hypsizygus marmoreus*, the greatest equivalent umami concentration (EUC) was observed after hot-air drying at 70 °C in the pileus and 60 °C in the stipe. Elevated EUC values were associated primarily with higher levels of 5′- guanosine monophosphate (5′-GMP), glutamic acid, succinic acid and citric acid [44]. Interestingly, elevated hot-air drying temperatures may improve the retention of certain aroma-related properties. In *Boletus edulis*, convective drying at 70–80 °C preserved relatively high concentrations of volatile compounds and favourable sensory characteristics, whereas FD remained the most effective method for achieving overall product quality [39]. Hot air drying was also efficient in terms of processing time and showed good preservation of phenolic compounds and antioxidant activity in some applications [22]. Studies consistently show that high drying temperatures (around or above 80 °C) can degrade flavour compounds, reduce nutritional quality and induce undesirable colour changes [37,45]. For *Agaricus bisporus*, convective drying at 60 °C harmed colour, antioxidant activity, rehydration capacity and texture compared to 40 °C and 50 °C [45].

#### 3.4.3. Vacuum and Microwave-Assisted Drying

Vacuum drying and microwave-assisted drying can improve the retention of quality attributes compared to conventional hot-air drying while reducing drying time and potentially lowering energy consumption [37,46,47]. These methods reduce drying temperature and drying time by enhancing heat and mass transfer during moisture removal [23,47]. Vacuum drying is performed under reduced pressure conditions, which decrease the boiling point of water and allow moisture removal at comparatively low temperatures. Such conditions are advantageous for retaining thermolabile and bioactive compounds. Studies on *Cantharellus cibarius* (chanterelle mushrooms) have therefore explored the optimization of vacuum drying parameters to improve the physicochemical quality of the final dried product [47]. By combining microwave heating with vacuum conditions, microwave vacuum drying accelerates water evaporation and can improve heating uniformity compared with conventional drying methods [23]. However, research on *Boletus edulis* showed that VMD produced less favourable sensory characteristics compared with the other drying methods evaluated, indicating that careful optimization of processing parameters is necessary [39]. Advanced hybrid drying strategies have attracted increasing attention due to their potential to improve product quality and process efficiency. Intermittent microwave vacuum drying alternates microwave heating with resting periods, which can reduce localized overheating and help preserve structural integrity during drying. Similarly, combined hot air–microwave drying has demonstrated promising results in *Pleurotus eryngii*, achieving the highest amino acid content (560.94 mg/100 g) and sensory evaluation score (9.4 points), together with favourable shrinkage (55.43%), rehydration capacity (1.83), colour value (76.29), and polysaccharide content (11.8%) [23].

#### 3.4.4. Heat Pump Dehumidifier Drying

Heat pump dehumidifier drying (HPD) has gained attention as an energy-efficient drying approach for mushrooms because it can maintain high sensory and flavour quality while reducing energy demand compared with conventional drying methods. In shiitake mushrooms, HPD enhanced characteristic flavour compounds and umami intensity, achieved superior overall sensory quality and required considerably lower energy consumption (0.85 kWh/kg) than hot air drying and VFD [3].

#### 3.4.5. Centrifugal Vacuum Drying

Centrifugal vacuum drying (CVD) is an emerging technique that combines vacuum conditions with centrifugation and has recently been investigated as an alternative mushroom drying method. In *Boletus edulis*, CVD demonstrated good preservation of bioactive compounds, colour characteristics and thermal stability, whereas FD resulted in superior rehydration capacity and better microstructural preservation. However, FD required substantially longer processing times (approximately 19 h), indicating that CVD could represent a promising and more practical alternative for mushroom processing applications [22]. To enhance drying quality, various pretreatments have been investigated to improve drying efficiency and product quality [22,24,42]. Cold plasma pretreatment prior to hot-air drying has emerged as a promising strategy due to its ability to alter surface microstructure, enhance moisture transfer, shorten drying time, and improve the retention of nutritional compounds [24,27]. Compared with water blanching, Ultraviolet C (UV-C) pretreatment resulted in better retention of phenolic compounds and antioxidant activity while causing smaller colour alterations [22]. Osmotic pretreatments using salt solutions have also been investigated for *Pleurotus ostreatus*, with higher salt concentrations leading to lower protein, fat, and fibre contents, alongside increases in ash and carbohydrate levels [42]. Vacuum impregnation has recently been explored as a strategy for calcium enrichment of *Agaricus bisporus*. Using this approach, calcium levels increased up to 76 times compared to the initial value after only 10 min of treatment. In addition, the freezing technique applied before FD affected product quality. Samples subjected to vacuum freezing showed faster FD kinetics and better overall quality characteristics than those frozen by conventional contact freezing [45]. These findings suggest that optimization of pretreatment and freezing conditions may play an important role in improving preservation outcomes.

### 3.5. Edible Coatings

Edible coatings represent a minimally invasive preservation strategy that forms a semi-permeable barrier on mushroom surfaces, helping regulate gas exchange, reduce moisture loss and facilitate the incorporation of antimicrobial and antioxidant compounds [31,32]. Recent years have witnessed significant advances in edible coating formulations, application strategies, and the understanding of mechanisms involved in mushroom preservation [28].

#### 3.5.1. Polysaccharide-Based Coatings

Polysaccharide-based edible coatings, particularly chitosan, sodium alginate, guar gum, and agar-derived materials, have been widely investigated for their ability to maintain mushroom quality and extend shelf life [28,31,48]. These biopolymers are valued for their film-forming capacity, biodegradability and potential antimicrobial properties [28]. Sodium alginate-based edible composite coatings have demonstrated promising potential for preserving *Pholiota nameko* mushrooms by improving postharvest quality and delaying deterioration processes [49]. For *Agaricus bisporus*, various edible coatings based on polysaccharides and proteins, including gum, agar, sodium alginate, egg white protein and lecithin, have been investigated for postharvest preservation, with sodium alginate showing particularly strong efficacy in reducing weight loss and browning [32]. Chitosan-based coatings contribute to mushroom preservation through several mechanisms, including antimicrobial activity, antioxidant effects associated with reduced enzymatic browning and barrier properties that limit moisture loss and gas exchange [28]. Research on *Lentinus edodes* showed that composite coatings based on chitosan and guar gum effectively preserved postharvest quality, with coating performance depending on the guar gum concentration used in the formulation [31]. Recent studies have shown that modification strategies and functional additives can improve the barrier, antioxidant and antimicrobial properties of polysaccharide-based coatings. Essential oils and polyphenolic compounds have been incorporated into polysaccharide-based coatings to enhance their antimicrobial and antioxidant properties, while modification strategies can improve water and gas barrier characteristics and reduce light transmittance [28].

#### 3.5.2. Lipid-Based and Composite Coatings

Lipid-based edible coatings are considered effective moisture barriers because their hydrophobic structure can reduce water loss during storage. In *Pleurotus ostreatus*, GMS coatings supplemented with *Thymus vulgaris* extract, particularly at 150 mg/kg, improved firmness, limited weight loss, and reduced changes in colour parameters throughout storage. The combined treatment also inhibited microbial growth and contributed to better preservation of mushroom appearance, while the GMS layer acted as a physical barrier against moisture and oxygen transfer [33]. Composite coatings combining polysaccharides with lipids or proteins can provide complementary functional properties, improving moisture retention, gas barrier performance, and overall postharvest quality preservation [31,32]. Chitosan–guar gum composite coatings combine the antimicrobial properties of chitosan with the stabilizing and film-forming functions of guar gum, contributing to improved postharvest preservation [31].

#### 3.5.3. Active Coatings with Antimicrobial Agents

Active edible coatings incorporating antimicrobial and antioxidant compounds represent an advanced preservation strategy that combines physical barrier functions with bioactive protection mechanisms [33,34]. Plant extracts, essential oils and other natural antimicrobials can be incorporated into coating matrices to enable the sustained release of bioactive compounds with antimicrobial and antioxidant activity [28,33]. Research on button mushrooms showed that combining an active edible coating based on *Salvia macrosiphon* seed gum enriched with liquid smoke with UV-B irradiation effectively delayed browning, reduced microbial growth, preserved texture and extended shelf life during refrigerated storage. The preservation effect was associated with the antimicrobial and antioxidant properties of the coating components, together with the ability of UV-B treatment to maintain postharvest quality. The combined treatment provided better overall preservation compared with untreated samples and contributed to improved sensory and physicochemical stability during storage [34]. Microbiological assessment of white button mushrooms coated with edible films has demonstrated that properly formulated coatings can contribute to shelf-life extension by reducing moisture loss, delaying browning, and preserving texture. Although antimicrobial efficacy depends on coating composition and processing conditions, active coatings remain a promising strategy for maintaining the quality and stability of fresh mushrooms during storage [50].

### 3.6. Fermentation and Lactic Acid Biopreservation

Fermentation represents a traditional preservation method that has gained renewed interest for mushroom applications, particularly in the valorization of mushroom by-products and the development of value-added products with enhanced bioactive, antioxidant, and functional properties [18]. Selected lactic acid bacteria can contribute to mushroom preservation through acidification, antimicrobial metabolite production, enhancement of antioxidant and bioactive properties, and the development of characteristic volatile compounds during fermentation [18,21]. Fermentation is increasingly recognized as a form of biopreservation because the metabolic activity of lactic acid bacteria can suppress spoilage microorganisms, improve microbial safety, and enhance product stability during storage. Similar LAB-based preservation approaches have been widely applied in fermented vegetables and cereal-based products, where fermentation contributes not only to shelf-life extension but also to improved nutritional and functional quality. Consequently, mushroom fermentation can be considered a modern preservation strategy that combines biopreservation with the development of functional food products [21,51].

### Lactic Acid Bacteria Fermentation

Studies on *Hericium erinaceus* by-products showed that fermentation with *Lactoplantibacillus plantarum* and *Lacticaseibacillus rhamnosus* improved microbial stability by reducing pH and inhibiting spoilage microorganisms, including yeasts, fungi and *Pseudomonas* spp. Fermentation also enhanced the functional properties of the substrate through increased total phenolic content and antioxidant activity, while limiting the accumulation of harmful biogenic amines such as cadaverine. In addition, the process reduced gel strength and promoted a softer, more liquid-like texture, which could be advantageous for the development of semi-liquid or processed functional foods [18].

### 3.7. Irradiation Technologies

Non-thermal irradiation methods such as gamma rays, electron beams, and ultraviolet (UV) treatment have been widely investigated as preservation technologies due to their ability to reduce microbial contamination while largely preserving the sensory and nutritional quality of food products. Recent research has primarily focused on optimizing irradiation parameters, evaluating their effects on quality attributes and bioactive compounds, and developing reliable detection methods for irradiated food products [17,52].

#### 3.7.1. Electron Beam Irradiation

Yoon et al. (2024) [17] demonstrated that electron beam irradiation (EBI) effectively preserved the quality of *Pleurotus eryngii* by reducing microbial contamination and browning while maintaining moisture content, water activity, and firmness at doses up to 2 kGy. The study also confirmed that photostimulated luminescence (PSL) and thermoluminescence (TL)methods can successfully detect irradiated mushroom products.

#### 3.7.2. Gamma and UV Irradiation

Gamma and UV irradiation are widely recognized preservation technologies that effectively maintain mushroom quality and extend shelf life by reducing microbial growth and delaying postharvest deterioration. Recent advances in postharvest irradiation preservation technology have highlighted significant progress in extending the shelf life and maintaining the quality of edible fungi through different irradiation approaches [52]. Combined irradiation and refrigeration treatments have been shown to improve mushroom preservation by reducing microbial spoilage, delaying postharvest maturation, and maintaining quality attributes during storage. The integration of electron beam irradiation, cooling and modified atmosphere conditions extended mushroom shelf life up to 20 days while preserving microbiological and organoleptic quality [53]. UV-C pretreatment prior to drying was shown to better preserve phenolic compounds and antioxidant activity compared to conventional hot water blanching [22]. UV-B irradiation combined with active edible coatings demonstrated synergistic effects in preserving the quality and extending the shelf life of button mushrooms [34]. Overall, these findings indicate that UV-based technologies represent versatile and promising approaches for mushroom preservation, either as independent treatments or in combination with other preservation techniques within integrated postharvest strategies [22,34]. A comparative overview of the major preservation technologies applied to edible and medicinal mushrooms, including their principal effects, advantages, limitations and tested species, is presented in Table 1.

## 4. Comparative Analysis of Preservation Efficacy

Comparative analyses of mushroom preservation methods demonstrate notable differences in preservation efficiency, quality retention, energy demand and economic feasibility [19,20,37]. The selection of an appropriate preservation method depends on factors such as mushroom species, intended use, desired quality characteristics, shelf-life requirements and economic feasibility [4,14,19].

### 4.1. Drying Method Comparisons

Comparative studies involving multiple mushroom species provide valuable insights into the relative performance of different drying methods [3,39,41]. Among the evaluated drying techniques for *Stropharia rugosoannulata*, vacuum freeze drying (VFD) resulted in the highest overall product quality. Compared with hot-air, solar, room-temperature shaded, and microwave drying, VFD better preserved mushroom morphology and colour while minimizing shrinkage. In addition, VFD samples retained higher levels of proteins, carbohydrates, total sugars, vitamin C and bioactive compounds, which was associated with enhanced antioxidant activity [36]. For shiitake mushrooms (*Lentinus edodes*), HPD was identified as an effective compromise between product quality and energy efficiency. Compared with hot-air drying and vacuum freeze drying, HPD enhanced characteristic mushroom flavour through higher volatile sulphide content and increased equivalent umami concentration. In addition, HPD samples exhibited intermediate shrinkage, rehydration capacity, and microstructural properties while achieving the highest overall sensory quality. Importantly, HPD required substantially lower energy input (0.85 kWh/kg) than HAD (2.65 kWh/kg) and VFD (6.67 kWh/kg), corresponding to approximately 87% lower energy consumption than freeze drying [3]. In *Pleurotus eryngii*, VFD preserved structural integrity most effectively, resulting in the lowest shrinkage rate (30.2%), highest rehydration ratio (3.03) and greatest polysaccharide retention (13%). In contrast, hot air–microwave vacuum combined drying produced the highest amino acid content (560.94 mg/100 g) and achieved the best sensory evaluation score (9.4). Hot air drying alone produced the lowest polysaccharide retention (5.4%) and the lowest sensory score (4.4 points), further supporting the advantages of combined drying techniques [23]. For *Boletus edulis*, FD provided the best overall sensory quality, whereas convective drying at 70 °C and 80 °C resulted in the highest retention of volatile compounds and key positive mushroom aroma attributes. Vacuum microwave drying showed the least favourable sensory profile, while the combined convective predrying and vacuum microwave finish-drying approach yielded intermediate retention of volatile compounds and sensory attributes [39].

### 4.2. Fresh Preservation Method Comparisons

For fresh mushroom preservation, comparative analysis reveals distinct advantages and limitations of different approaches [10,15,16]. Cold plasma and plasma-activated water treatments demonstrated promising preservation effects in *Agaricus bisporus*. Plasma-activated water reduced browning index and PPO activity, decreasing PPO activity from 1788 U/min in the control group to 228 U/min after 5 days of storage. In addition, treated mushrooms exhibited improved tissue firmness (19.69 N) and lower weight loss (0.15% after 3 days), indicating enhanced postharvest quality and storage stability [15]. The combination of LVEF and MAP demonstrated synergistic preservation effects in *Agaricus bisporus*, extending storage stability at 4 °C from approximately 6 to more than 12 days. Compared with individual treatments, the combined approach more effectively reduced respiration and microbial proliferation, modulated enzyme-related deterioration processes and preserved cellular membrane integrity, resulting in improved postharvest quality [10]. Among the evaluated modified atmosphere packaging systems, HCP provided the most effective preservation of *Pleurotus sapidus* quality during refrigerated storage. After 10 days, HCP-treated samples retained the highest total phenolic content (approximately 2.85–3.03 mg GAE/g) and exhibited the highest lightness value (L* = 60.36), indicating reduced browning and improved colour stability. In addition, HCP achieved the lowest Total Quality Index values, with scores of 3.44 on day 5 and 4.74 on day 10, reflecting superior overall postharvest quality compared with the other MAP treatments [16].

### 4.3. Energy Efficiency and Economic Considerations

Energy consumption represents a major determinant of the economic and commercial sustainability of preservation technologies, particularly in large-scale industrial application [3,39,41]. Freeze-drying, despite superior quality retention, requires 6.67 kWh/kg for shiitake mushrooms, making it economically feasible primarily for high-value products [3]. In comparison, hot air drying consumes 2.65 kWh/kg, whereas heat pump dehumidifier drying demonstrates considerably lower energy consumption at 0.85 kWh/kg [19]. For *Lyophyllum decastes*, vacuum freeze drying exhibited the longest drying time (approximately 22 h) and the highest energy consumption, whereas hot air drying was identified as the most energy-efficient method. The trade-off between product quality and energy consumption necessitates careful consideration of the target market, product positioning, and economic constraints when selecting an appropriate drying method [41]. Non-thermal preservation technologies, including cold plasma, electrostatic processing and active packaging systems, offer promising alternatives to conventional thermal treatments by reducing quality deterioration while potentially lowering energy requirements. However, their industrial applicability may still be influenced by equipment costs and operational complexity [10,15]. The integration of multiple preservation strategies may offer an optimal balance between quality, shelf life and economic viability [10,34].

## 5. Quality Parameters and Nutritional Retention

Different preservation technologies influence critical quality parameters of mushrooms, including colour stability, texture, flavour profile, nutritional value and the preservation of bioactive constituents [17,36,40]. Preservation methods exert distinct effects on key quality attributes, including colour, texture, flavour, nutritional composition and the retention of bioactive compounds [4,14,19].

### 5.1. Colour Retention

Colour represents one of the most important quality attributes influencing consumer acceptance and purchasing behaviour [11,39,40]. Enzymatic browning, primarily mediated by PPO and tyrosinase, leads to undesirable darkening and substantial reductions in mushroom marketability [11,15]. FD consistently demonstrates superior colour retention across multiple mushroom species. In *Stropharia rugosoannulata*, optimized VFD produced samples with bright colour, minimal browning and retention of the natural white appearance after drying [40]. For *Oudemansiella raphanipes*, VFD achieved the highest L* value (67.09), indicating superior colour retention compared to other drying methods [37]. For *Lyophyllum decastes*, VFD provided the best colour retention among the tested drying methods, showing the highest L* value and the lowest colour difference [41]. Cold plasma and plasma-activated water treatments have shown strong potential for inhibiting enzymatic browning in mushrooms. In *Agaricus bisporus*, cold plasma treatment reduced browning by 26.9% compared with the control [11]. Plasma-activated water treatment effectively suppressed enzymatic browning in *Agaricus bisporus* by reducing BI and ΔE values and inhibiting PPO activity from 1788 U/min in untreated samples to 228 U/min after 5 days of storage [15]. Drying temperature is an important factor influencing colour stability during thermal processing. In *Agaricus bisporus*, samples dried convectively at 60 °C exhibited greater colour deterioration than those processed at 40 °C or 50 °C [45]. In *Pleurotus sajor-caju*, microwave-dried samples exhibited the greatest colour change, whereas freeze-dried samples showed the lowest degree of colour deterioration [20].

### 5.2. Texture and Rehydration Properties

Maintaining textural quality is essential for consumer acceptance and overall quality of both fresh and dried mushroom products [15,36,41]. In fresh mushrooms, firmness loss is mainly associated with cell wall degradation, moisture loss, and enzymatic activity during storage [11,15]. PAW treatment effectively preserved the firmness of *Agaricus bisporus*, maintaining tissue firmness at 19.69 N after 5 days of storage [15]. Cold plasma treatment effectively preserved mushroom firmness, maintaining hardness values 25.6% higher than those of the control samples [11]. Electron beam irradiation at doses up to 2 kGy preserved the firmness of *Pleurotus eryngii* without adverse effects [17]. In dried mushrooms, rehydration capacity is commonly used as an indicator of structural integrity and overall product quality [20,41]. Freeze-dried samples consistently show the highest rehydration ratios due to better preservation of porous microstructure and cellular integrity. In *Pleurotus eryngii*, vacuum freeze-drying achieved the highest rehydration ratio (3.03) [23]. In *Pleurotus sajor-caju*, freeze-dried samples exhibited greater rehydration capacity than samples dried by hot-air or microwave methods [20]. In *Lyophyllum decastes*, vacuum freeze drying resulted in the greatest rehydration capacity, with a rehydration ratio of 5.66 [41]. Shrinkage during drying inversely correlates with rehydration capacity. For *Pleurotus eryngii*, VFD achieved the smallest shrinkage (30.2%), while hot air drying resulted in greater shrinkage [23]. Microstructural analysis demonstrated that FD preserves the porous cellular structure more effectively than thermal drying methods, thereby enhancing water absorption and improving rehydration capacity [22,23,41].

### 5.3. Nutritional Content and Bioactive Compounds

Maintaining nutritional quality and bioactive compounds during preservation is essential for retaining the functional food value of mushrooms [21,22,24]. Heat-sensitive constituents, such as vitamins, phenolic compounds, and certain proteins, are especially susceptible to thermal degradation during conventional drying processes [19,22,24]. Freeze-drying demonstrates superior retention of heat-sensitive nutrients. In *Stropharia rugosoannulata*, VFD retained higher levels of protein, carbohydrates, total sugars and vitamin C compared to other drying methods [36]. In *Oudemansiella raphanipes*, VFD resulted in the highest 5′-nucleotide content (2.44 mg/g), while VMD yielded the highest crude protein content (26.13 mg/g dry weight) [37]. Cold plasma pretreatment prior to hot-air drying preserved higher levels of nutritional attributes, including sugars, vitamins and phenolic acids, while maintaining greater antioxidant activity compared to conventional hot-air drying without pretreatment. These findings highlight the potential of pretreatment strategies to mitigate thermal degradation during subsequent drying processes [24]. Fermentation can enhance bioactive compound content. For *Hericium erinaceus* by-products, lactic acid fermentation increased total phenolic content from 32 to 48 mg GAE/100 g, and antioxidant activity reached 2562 µmol TE/100 g [18]. In wild mushrooms, LAB fermentation combined with suitable pre-treatments improved product safety while enhancing volatile and bioactive compound profiles [21]. Polysaccharide content, which is closely associated with immunomodulatory and other bioactive properties, was strongly influenced by the preservation method. In *Pleurotus eryngii*, VFD preserved the highest polysaccharide content (13%), whereas hot-air drying resulted in the lowest levels (5.4%) [23]. For *Stropharia rugosoannulata*, optimized VFD better preserved bioactive substances and maintained stronger antioxidant activity [36,40].

### 5.4. Flavour and Volatile Compounds

Flavour quality, determined by volatile compounds and non-volatile taste components, influences consumer acceptance and culinary applications [3,39,44]. Preservation methods differentially influence volatile compound profiles through mechanisms involving thermal degradation, enzymatic activity and Maillard reactions [3,39,46]. Heat pump dehumidifier drying improved the characteristic flavour of shiitake mushrooms by increasing volatile sulphide levels and equivalent umami concentration while partially inhibiting excessive enzymatic and Maillard reactions [3]. In *Boletus edulis*, convective drying at 70 °C and 80 °C preserved high levels of volatile compounds together with favourable sensory characteristics [39]. Hot air drying promoted more intense aroma with higher aldehyde content (32.87%) for *Lyophyllum decastes*, while VFD retained flavour more similar to fresh samples [41]. In *Hypsizygus marmoreus*, hot air drying (HAD) at 70 °C for the pileus and 60 °C for the stipe resulted in the highest EUC. Higher EUC values were associated with elevated levels of 5′-GMP, succinic acid, citric acid, and glutamic acid [44]. For *Oudemansiella raphanipes*, ultrasound-assisted hot air drying at 60 °C yielded the highest free amino acid content (83.78 mg/g) and equivalent umami concentration (1491.33 MSG/100 g). High drying temperatures generally degrade flavour compounds. Studies have shown that drying above 80 °C reduces free amino acids and 5′-nucleotides, decreases umami intensity, and promotes Maillard reactions associated with off-flavour development [37]. Metabolomic analysis of *Dictyophora rubrovolvata* revealed that drying temperature influenced metabolite composition, with FD best preserving heat-sensitive and antioxidant-related compounds [46].

## 6. Discussion

**Figure 5:**
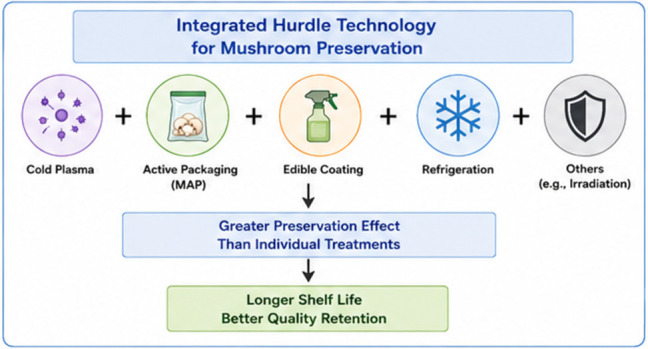
Integrated hurdle technology concept.

Recent research indicates that integrated preservation approaches provide superior quality retention and shelf-life extension compared with single preservation methods [10,33,34]. Combined preservation approaches simultaneously target multiple deterioration mechanisms, including enzymatic browning, microbial growth, moisture loss and respiratory metabolism, while enabling lower treatment intensity and improved retention of product quality [10,34]. The combination of low-voltage electrostatic field and modified atmosphere packaging (LVEF-MAP) represents an effective hurdle technology (Figure 5), extending the shelf life of *Agaricus bisporus* from approximately 6 to more than 12 days by reducing respiration intensity, inhibiting microbial growth, and maintaining membrane integrity [10]. The synergistic effect of LVEF–MAP is attributed to the complementary action of both technologies. LVEF suppresses respiration, microbial growth, and enzyme activity, whereas MAP reduces water loss and modifies the storage atmosphere. As a result, the combined treatment targets multiple deterioration pathways and extends mushroom shelf life more effectively than either technology alone [10]. Similarly, active edible coatings combined with UV-B irradiation have demonstrated synergistic antimicrobial and antioxidant effects while preserving texture and sensory properties during storage [34]. Cold plasma pretreatment prior to drying improves drying efficiency and enhances the retention of heat-sensitive bioactive compounds compared with conventional hot-air drying alone [24,27]. Among the available drying technologies, FD is generally considered the most effective method for preserving product quality, particularly colour, texture, rehydration capacity and bioactive compounds [3,41]. Despite its superior product quality, FD remains limited in large-scale industrial applications due to high energy demand, prolonged processing times, and elevated operational costs. Heat pump dehumidifier drying has emerged as a promising alternative to FD, providing a favourable balance between product quality and energy efficiency [3]. Despite substantial technological advances, several challenges still limit large-scale implementation of modern mushroom preservation technologies [4,9,14,19]. Preservation efficacy is highly species-dependent, requiring optimization of processing parameters according to mushroom species, maturity stage, and targeted quality attributes [3,20,23,39]. Preservation efficacy is also influenced by biological factors, including mushroom species, harvesting maturity and the composition of associated microbial communities. Variations in physiological characteristics and microbial interactions may affect postharvest responses to preservation treatments. Consequently, preservation parameters optimized for one mushroom species or production system may not necessarily be directly applicable to others, highlighting the need for species-specific optimization and validation [54]. In addition, the lack of standardized analytical methodologies and quality evaluation criteria hinders direct comparison among studies [4,19]. Commercial implementation of emerging technologies such as cold plasma, electrostatic processing and active packaging is further constrained by high equipment costs, technical complexity, and regulatory challenges [9,11,15]. Despite the promising results reported for active packaging systems and atmospheric-pressure cold plasma, several food safety considerations remain. Packaging materials may release low-molecular-weight compounds into food, and the extent of migration depends on factors such as temperature, storage duration, food composition, and packaging characteristics. These substances may affect food quality and, in some cases, raise concerns regarding consumer exposure. In addition, although atmospheric-pressure cold plasma is considered an effective non-thermal preservation technology, its antimicrobial activity is associated with the generation of reactive oxygen and nitrogen species. Therefore, optimization of treatment conditions and further safety evaluations are necessary to ensure that preservation benefits are achieved without compromising product quality or consumer safety [55,56]. Despite promising preservation efficacy, many emerging technologies are currently confined to laboratory and pilot-scale studies, whereas continuous industrial-scale processing systems remain underdeveloped [4,14]. Several important research gaps remain regarding the application of emerging non-thermal preservation technologies for mushrooms [4,34]. Long-term storage stability, consumer acceptance, sensory evaluation and environmental sustainability assessments remain underrepresented in the current literature [1,3,9]. Furthermore, limited information is available regarding the influence of preservation technologies on medicinally important compounds such as ganoderic acids, triterpenoids and cordycepin. Most studies primarily evaluate preservation efficacy through physicochemical, microbiological, and sensory parameters, whereas the retention of pharmacologically active compounds receives considerably less attention. Future research should therefore assess preservation performance not only in terms of product quality but also with respect to the stability and bioavailability of medicinally relevant constituents. Furthermore, most preservation studies have focused on cultivated mushroom species, whereas information regarding wild medicinal fungi remains limited. Differences in physiological characteristics, storage tolerance and susceptibility to quality deterioration may influence preservation outcomes. Therefore, additional studies on wild medicinal fungi are needed to support the development of targeted preservation strategies. At present, several preservation technologies, including modified atmosphere packaging (MAP), conventional drying methods, freeze-drying, and selected edible coating systems, have reached commercial or semi-commercial implementation in the mushroom industry. In contrast, emerging technologies such as cold plasma treatment, electrostatic field processing, antimicrobial photodynamic therapy and nanocomposite-based active packaging remain largely at the laboratory or pilot scale. Although these innovative approaches demonstrate promising preservation efficacy, further research is required to establish standardized operating conditions, economic feasibility, regulatory compliance and large-scale industrial applicability.

As shown in Table 2, no single preservation technology effectively controls all deterioration pathways. Therefore, preservation strategies that combine complementary technologies are increasingly regarded as the most promising approach for maintaining mushroom quality and extending shelf life.

## 7. Future Research Directions

Future studies should focus on optimizing and facilitating the industrial application of non-thermal preservation technologies, particularly cold plasma, pulsed electric field, high-pressure processing, and antimicrobial photodynamic therapy [4,11,14,15]. Additional research is needed to optimize treatment parameters, evaluate long-term storage stability and investigate the effects of these preservation technologies on bioactive compounds and sensory attributes. Integrated hurdle technologies appear to be among the most promising strategies for future mushroom preservation [10,33,34]. Systematic investigation of synergistic preservation strategies, including the integration of cold plasma with drying technologies, electrostatic processing with active packaging systems and edible coatings enriched with antimicrobial agents, may improve preservation efficacy while reducing processing intensity and energy consumption [9,24]. Sustainability assessment should become a major component of future research. Life cycle analysis, carbon footprint evaluation, water consumption and waste generation associated with preservation technologies remain insufficiently investigated [3,9]. Development of biodegradable packaging materials and energy-efficient drying systems should therefore remain research priorities. Future studies should also focus on preservation approaches capable of enhancing, rather than only maintaining, bioactive compounds and functional properties [18,21]. Fermentation, controlled stress treatments and advanced encapsulation technologies may contribute to the development of value-added functional mushroom products. Finally, standardized quality evaluation protocols and consumer acceptance studies are necessary to facilitate the commercialization and market adoption of novel preservation technologies [3,4,14].

## 8. Conclusions

Edible and medicinal mushrooms remain highly perishable commodities, requiring effective preservation strategies to maintain quality, nutritional value, and bioactive compounds. Among the reviewed technologies, cold plasma treatment appears particularly suitable for fresh mushroom preservation due to its ability to reduce microbial contamination and enzymatic browning while maintaining texture and nutritional quality. Active packaging systems and edible coatings are effective options for refrigerated storage, whereas freeze-drying remains the preferred method for dried mushroom products because of its superior retention of colour, texture, and bioactive compounds. Despite their proven effectiveness, the widespread industrial application of these technologies remains limited by high equipment costs, energy requirements, insufficient process standardization, and a lack of large-scale validation studies. These factors currently represent the main bottlenecks preventing broader commercial adoption. Current evidence indicates that integrated preservation approaches combining multiple technologies offer the greatest potential for future development. In particular, combinations such as electrostatic field treatment with modified atmosphere packaging, active coatings with UV treatment, and cold plasma-assisted preservation systems have demonstrated synergistic effects on shelf-life extension and quality retention. Future research should therefore focus on the development of standardized, economically feasible, and energy-efficient hurdle technologies suitable for industrial implementation [3,9,10,14,15,18,28,29,36,41,46,48].

## Figures and Tables

**Table 1:** Comparative overview of emerging preservation technologies for edible and medicinal mushrooms.

Technology	Main Effect	Typical Shelf-Life Extension/Quality Retention	Industrial Maturity	Advantages	Limitations	References
Cold plasma treatment	Reduces microbial load, enzymatic browning and PPO activity	PPO activity reduced from 1788 to 228 U/min (~87% reduction); browning reduced by26.9%; firmness increased by 25.6%	Pilot scale	Non-thermal preservation; maintains firmness, vitamin C, proteins and antioxidant activity	High equipment cost and limited industrial-scale validation	[11,15,24,26,27]
Active packaging/MAP	Slows respiration, senescence and microbial growth	Shelf life commonly extended to 8–10 days under refrigeration; improved colour and phenolic retention	Commercial	Extends shelf life and preserves texture, colour and phenolic compounds	Gas composition and packaging materials require optimization	[9,16,29,30]
Electrostatic field + MAP	Reduces respiration intensity, water loss and enzyme activity	Shelf life extended from ~6 to >12 days	Pilot scale	Shelf-life extension from approximately 6 to >12 days	Requires specialized electrostatic equipment	[10]
Freeze-drying (lyophilization)	Preserves colour, texture, microstructure and bioactive compounds	Highest retention of colour, rehydration and bioactive compounds	Commercial	Highest quality retention and superior rehydration properties	High energy consumption, long drying time and high operational cost	[19,23,36,37,39,40,41]
Hot-air drying	Reduces water activity and enables long-term storage	Long-term stability but lower nutrient retention than FD	Commercial	Economical, simple and widely applied industrially	Thermal degradation of nutrients, colour and volatile compounds	[20,39,43,44]
Vacuum and microwave-assisted drying	Accelerates drying and improves heat and mass transfer	Improved nutrient retention and shorter drying time than HAD	Semi- commercial	Shorter drying time and improved retention of nutrients compared with conventional HAD	Possible uneven heating and quality deterioration if parameters are not optimized	[22,23,37,47]
Heat pump dehumidifier drying	Energy-efficient dehydration and flavour preservation	~87% lower energy consumption than FD (0.85 vs. 6.67 kWh/kg)	Commercial	Lower energy consumption with acceptable sensory and physicochemical quality	Slightly lower quality retention than freeze-drying	[3]
Edible coatings (polysaccharide-, lipid- and composite-based)	Reduces moisture loss, browning and microbial growth	Reduced weight loss, browning and microbial growth	Commercial/ Semi- commercial	Biodegradable; can incorporate antimicrobial and antioxidant compounds	Preservation efficacy depends on coating formulation and storage conditions	[28,31,32,33,48,49,50]
Fermentation/biopreservation	Improves microbial stability and enhances bioactive properties	Increased phenolics (32 → 48 mg GAE/100 g) and antioxidant activity	Pilot scale	Increases phenolic compounds and antioxidant activity	May alter texture and flavour profile	[18,21]
Irradiation technologies (electron beam, UV and gamma irradiation)	Reduces microbial contamination and delays postharvest spoilage	Shelf life up to 20 days when combined with cooling/MAP; maintained firmness and microbiological quality	Commercial in some countries	Preserves physicochemical and sensory quality while extending shelf life	Regulatory limitations and consumer acceptance challenges	[17,22,34,52,53]

**Table 2:** Relationship between preservation objectives and suitable preservation technologies.

Preservation Objective	Main Deterioration Mechanism	Most Suitable Technologies	Typical Application Scenario	References
Browning control	PPO activity and oxidation of phenolic compounds	Cold plasma treatment, PAW, edible coatings	Fresh mushrooms during refrigerated storage	[11,15,32,34]
Microbial spoilage reduction	Growth of bacteria, yeasts and moulds	Cold plasma, irradiation, biopreservation (LAB fermentation)	Fresh mushrooms and minimally processed products	[11,17,18,21]
Moisture loss prevention	Transpiration and water evaporation	MAP, active packaging, edible coatings	Fresh mushrooms during storage and distribution	[9,10,28,32]
Respiration and senescence control	High metabolic activity and membrane degradation	MAP, electrostatic field treatment (LVEF/HVEF)	Refrigerated storage of fresh mushrooms	[10,16,35]
Retention of bioactive compounds	Oxidative and thermal degradation	Freeze-drying, cold plasma-assisted drying, fermentation	Functional and medicinal mushroom products	[3,18,24,27]
Long-term preservation	Reduction in water activity and microbial stability	Freeze-drying, HPD, vacuum-assisted drying	Dried mushroom products and ingredients	[3,22,36,37]
Integrated preservation (hurdle technology)	Multiple deterioration pathways simultaneously	LVEF–MAP, UV-B + active coatings, plasma-assisted drying	Advanced preservation systems	[10,24,27,34]

## Data Availability

The original contributions presented in this study are included in the article. Further inquiries can be directed to the corresponding author.

## Abbreviations

The following abbreviations are used in this manuscript:

PPO	Polyphenol Oxidase
MAP	Modified Atmosphere Packaging
LVEF	Low-Voltage Electrostatic Field
aPDT	Antimicrobial Photodynamic Therapy
FD	Freeze-Drying
HAD	Hot Air Drying
VFD	Vacuum Freeze-Drying
VMD	Vacuum Microwave Drying
HPD	Heat Pump Dehumidifier Drying
CVD	Centrifugal Vacuum Drying
ROS/RNS	Reactive Oxygen and Nitrogen Species
PAW	Plasma-Activated Water
DBD	Dielectric Barrier Discharge
HCP	High Carbon Dioxide Packaging
LCP	Low Carbon Dioxide Packaging
GMS	Glycerol Monostearate
UV-B	Ultraviolet B
UV-C	Ultraviolet C
EUC	Equivalent Umami Concentration
